# Peristomal skin and subcutaneous tissue metastasis of urothelial carcinoma and successful treatment with immunotherapy: A 5-year follow-up case report

**DOI:** 10.1016/j.eucr.2026.103642

**Published:** 2026-10-12

**Authors:** Fürkan Batuhan Güven, Hasan Soydan, Zeynep Tekdemir, Furkan Bingöloğlu, Ahmet Kenan Karademir

**Affiliations:** Department of Urology, Faculty of Medicine, Maltepe University, Maltepe, Istanbul, Türkiye

**Keywords:** Bladder cancer, Urothelial carcinoma, Metastasis, Cutaneous metastasis, Immunotherapy

## Abstract

Cutaneous metastasis of urothelial carcinoma is an extremely rare condition following muscle-invasive bladder cancer. Recent studies are highlighting the importance of immunotherapy. A 73-year-old patient presented to our clinic with muscle-invasive bladder cancer. Radical cystectomy was performed with the formation of ileal conduit. Six months after the adjuvant chemotherapy, the patient presented with peristomal skin and deep tissue metastasis. Intravenous immunotherapy (Atezolizumab) was applied for 14 cycles as a second-line treatment after metastasectomy. The patient remains disease-free after 5 years of follow-up without further medical intervention.

## 1 Introduction

Bladder cancer is the 9th most commonly diagnosed cancer worldwide^1^ and the second most common cancer of the genitourinary tract after prostate cancer. While both incidence and mortality rates are increasing due to its nature,^1^ approximately quarter of patients with bladder cancer present with muscle invasive tumor (T2-T4) with a higher risk of cancer-specific mortality compared to other variants.^1^, ^2^ As a treatment modality, radical cystectomy is the strongly suggested method with the following urinary diversion method of choice for the muscle invasive bladder cancer.^3^ Urinary diversion options include ureterostomy, ileal conduit or orthotopic neobladder reconstruction with ileal or ileocecal segments. As illustrated by our case, the ileal conduit continues to be the most widely utilized method of urinary diversion.^4^ The risk of disease relapse following radical cystectomy is reportedly 5–70%, with the majority occurring within 2 years of surgery.^5^, ^6^

Unfortunately, metastasis to surrounding tissue can occur following a radical cystectomy but metastasis specifically to the peristomal skin remains an extremely rare occurrence for invasive bladder cancer.^7^ Due to lack of evidence, managing such cases can be challenging. There is still a lack of established standards. However, there are case reports stating that excision,^8^ chemotherapy and immunotherapy^9^ are viable options.

In this case study, we present a patient with deep abdominal and peristomal skin metastasis after 6 months secondary to a high-grade, muscle-invasive urothelial carcinoma (MIBC). Moreover, while the peristomal tissue and skin were heavily involved and partially excised, the patient remains disease-free at five years follow-up after surgical removal of metastatic tissues and adjuvant immunotherapy.

## 2 Case presentation

In 2020, a 73-year-old patient presented to our clinic with painless hematuria. Abdominal ultrasonography revealed a 5 cm tumor in the bladder. Radiological evaluation with contrast enhanced computed tomography demonstrated that the tumor was originating from the left distal ureter than radiating inside to the bladder. Notably, there was no further involvement of the kidneys except hydronephrosis on the left side (Fig. 1, Fig. 2).

**Fig. 1:**
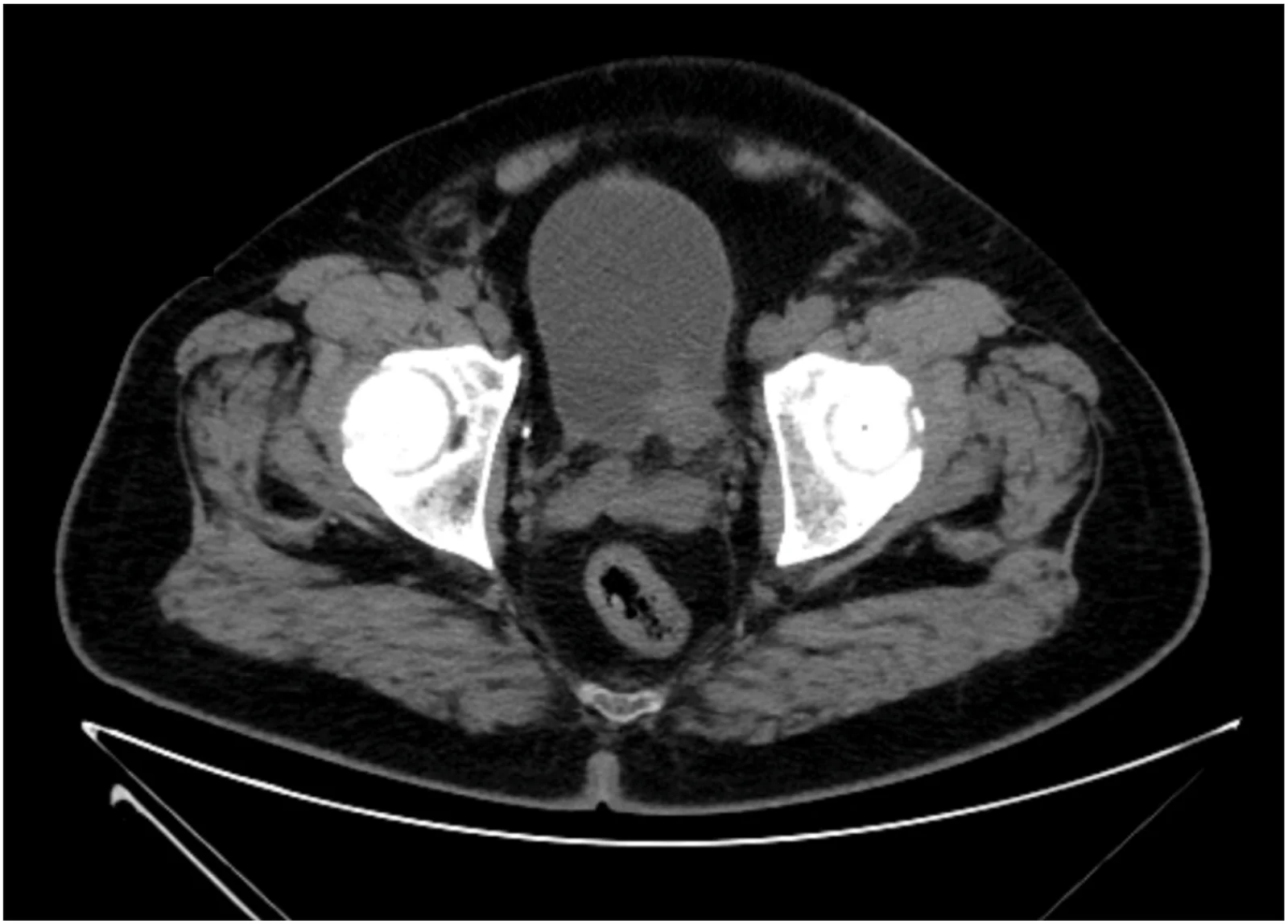
Non-contrast pelvic CT scan. Axial view demonstrating a soft-tissue mass along the posterolateral bladder wall protruding into the lumen.

**Fig. 2:**
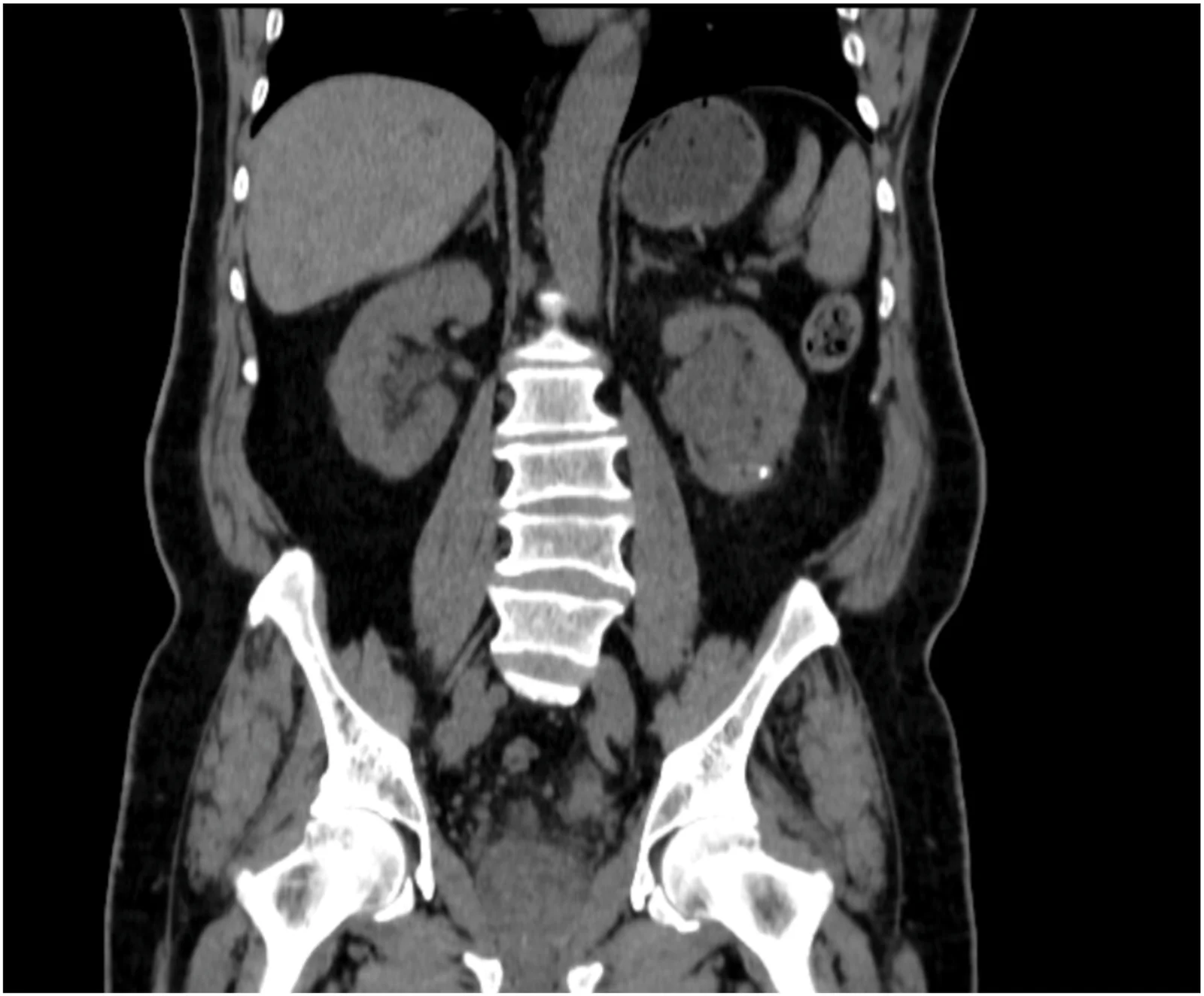
Non-contrast pelvic CT scan. Coronal view demonstrating left sided hydronephrosis and ill-defined tumor on the lower left side of the bladder.

During TUR-BT, a broad-based solid-papillary tumor was identified overlying the left ureteral orifice, with involvement of the adjacent detrusor muscle layers. Histopathological examination following incomplete resection demonstrated high-grade muscle-invasive urothelial carcinoma. After staging with FDG PET/CT demonstrated disease localized to the bladder and left ureter, the patient was scheduled to undergo radical cystectomy and ileal conduit urinary diversion. Because of the tumor involving the left ureter, a concomitant left nephroureterectomy was performed. Following radical cystectomy, ileal conduit urinary diversion was carried out in the standard fashion. Postoperative histopathological examination revealed urothelial carcinoma invading the renal pelvis and perirenal adipose tissue, as well as urothelial carcinoma involving the perivesical soft tissue and seminal vesicles. Metastatic involvement was identified in one iliac lymph node. Six cycles of adjuvant chemotherapy consisting of gemcitabine and cisplatin were administered for the treatment of urothelial carcinoma of the bladder and upper urinary tract urothelial carcinoma.

Six months after completing adjuvant therapy, the patient presented with gross hematuria and decreased urine output. Physical examination revealed an ulcerative tumor infiltrating the peristomal skin (Fig. 3). The lesion involved not only the local skin but also the underlying deep soft tissues. Notably, there was no evidence of invasion of the ileal conduit stoma itself. Intraoperative exploration and frozen section analysis revealed that metastasis was originating from urothelial carcinoma seeding from deep fascia and spreading to skin near stoma without the involvement of urinary diversion. After surgical partial excision of the tumor and peristomal skin tissue, evaluation with FDG PET/CT demonstrated no evidence of distant metastatic disease. Then adjuvant immunotherapy consisting of atezolizumab, was applied for 14 cycles as an alternative and second line therapy.

**Fig. 3:**
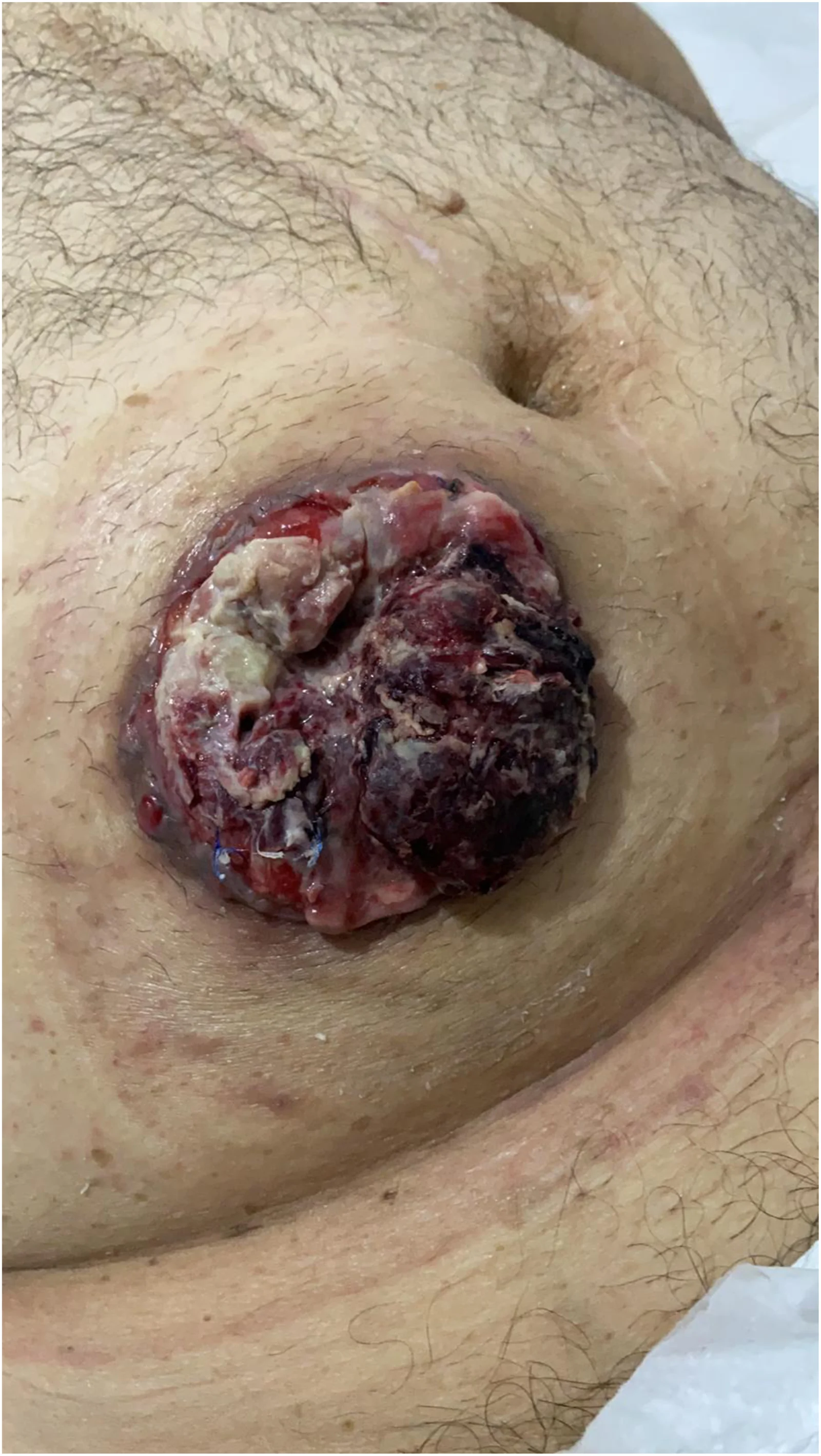
Preoperative clinical photograph. Exophytic cutaneous extension of a deep pelvic metastatic tumor, localized to the right lower abdominal wall near the umbilicus.

Following the immunotherapy treatment, the patient remains disease-free after 5 years of follow-up. No additional surgical or non-surgical treatment modality applied after adjuvant immunotherapy and no further management needed for ileal diversion (Fig. 4).

**Fig. 4:**
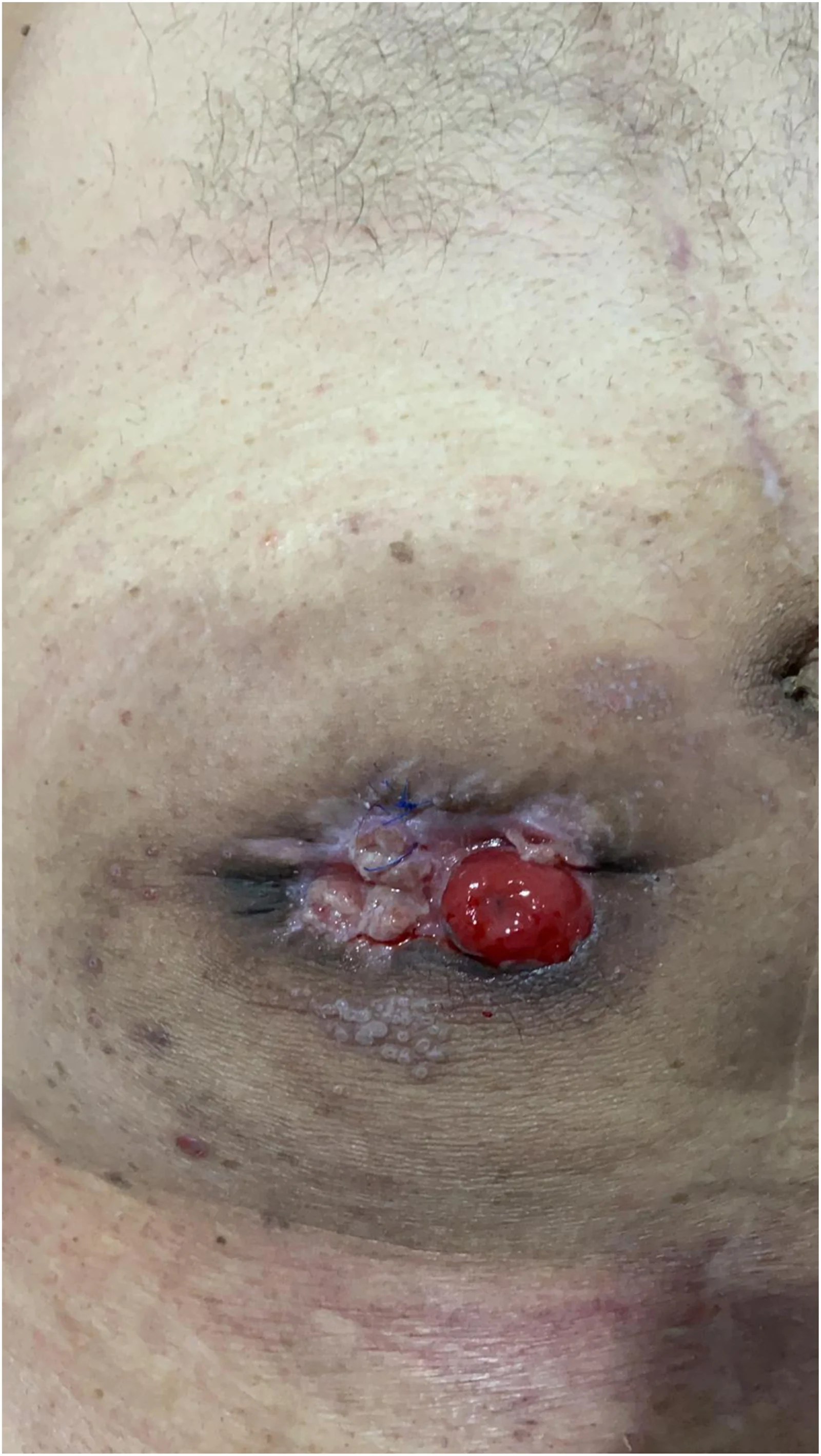
Postoperative clinical photograph. View of the lower right quadrant of the abdomen displaying the abdominal wall post-resection of the metastatic mass, alongside the preserved, intact medially located ileal conduit stoma.

## 3 Discussion

While the recurrence of invasive urothelial carcinoma is not deemed rare, skin involvement that is secondary to muscle-invasive bladder cancer (MIBC) appears to be an exceptionally rare phenomenon.^10^, ^11^ Most common sites for metastatic bladder cancer are expected to be regional lymph nodes, liver, lungs and bones.^12^ MIBC is generally documented to spread to distant tissues via hematogenous (bloodborne) or lymphatic pathways.^13^ Whereas, in our case the lesion originated from tumor seeding, direct or iatrogenic implantation.

Previous data demonstrate studies that involve metastatic management of urothelial carcinoma had mostly focused on chemotherapy as a first-line treatment whereas recent studies are highlighting the importance of immunotherapy.^14^ Nevertheless, after distant recurrence current guidelines offer excision, and systemic therapy and/or radiotherapy according to the location of the tumor and staging.^3^

In the treatment of locally invasive bladder cancer neoadjuvant cisplatin treatment may offer greater therapeutic efficacy, lesser side effects while prolonging survival time.^15^ On the other hand, new studies showed that immunotherapy for patients with advanced bladder cancer improves overall survival with a high response rate. Comparing immunotherapy with other treatment modalities, first-line immunotherapy for cisplatin-ineligible patients could be a favorable treatment option with exceptional results.^16^ Furthermore, combination of immunotherapy agent to frontline gemcitabine and cisplatin chemotherapy resulted better survival according to other researches^17^ and also case reports containing atezolizumab treatment can be recently found as a very effective treatment modality for urothelial cancer of the bladder.^18^ With these new approaches to disease management, immunotherapy may offer greater therapeutic efficacy while prolonging overall survival rate.^19^ Another study reported an unusual early regression of tumors after a short course of immunotherapy-based systemic treatment in patients with bladder tumors.^20^

In our case, the patient developed a local recurrence despite receiving adjuvant chemotherapy for locally advanced, lymph node–positive disease. As second-line treatment, immunotherapy with atezolizumab was initiated and continued for a total of 14 cycles via intravenous infusion. After five years of follow-up, the patient showed no signs of metastasis, local recurrence, or disease-related symptoms, resulting in long-term disease-free survival.

## 4 Conclusion

Although peristomal lesions are commonly seen after stoma formation, it is challenging to diagnose a lesion as ‘cutaneous infiltration secondary to urothelial carcinoma’ due to its extremely rare occurrence. With this case report, we demonstrate that the spread of the metastatic tumor could be controlled and treatment modalities including immunotherapy could extend the patient's disease-free interval to 5 years with appropriate surgical management. Despite the fact that this approach is new, experimental, and based on a single case, achieving five years of disease-free survival after metastatic spread suggests this method could be promising for selected patients with muscle-invasive bladder cancer. Still, to apply these findings in routine practice, validation in larger patient groups and prospective studies is needed.

## CRediT authorship contribution statement

**Fürkan Batuhan Güven:** Writing – original draft, Validation, Methodology, Investigation, Formal analysis, Data curation, Conceptualization. **Hasan Soydan:** Writing – review & editing, Validation, Supervision, Data curation. **Zeynep Tekdemir:** Writing – review & editing, Investigation, Data curation. **Furkan Bingöloğlu:** Validation, Supervision. **Ahmet Kenan Karademir:** Supervision.

## Ethics declaration

This study was performed in compliance with relevant laws, regulatory frameworks and guidelines where the research took place. Ethics committee approval was not required under relevant laws and institutional guidelines. Ethical approval was not required for this case report in accordance with the policies of our institution.

This research follows the CARE guidelines and the CARE checklist.

## Consent for publication

Written informed consent was obtained from the patient for publication of this case report and any accompanying images.

## Funding

This research did not receive any specific grant from funding agencies in the public, commercial, or not-for-profit sectors.

## Declaration of competing interest

The authors declare that they have no known competing financial interests or personal relationships that could have appeared to influence the work reported in this paper.
